# Machinability Investigation of Nitronic 60 Steel Turning Using SiAlON Ceramic Tools under Different Cooling/Lubrication Conditions

**DOI:** 10.3390/ma15072368

**Published:** 2022-03-23

**Authors:** Smita Padhan, Sudhansu Ranjan Das, Anshuman Das, Mohammad S. Alsoufi, Ahmed Mohamed Mahmoud Ibrahim, Ammar Elsheikh

**Affiliations:** 1 Department of Production Engineering, Veer Surendra Sai University of Technology, Burla 768018, India; smitapradhan3@gmail.com (S.P.); das.sudhansu83@gmail.com (S.R.D.); 2 Department of Mechanical Engineering, DIT University, Dehradun 248001, India; anshuman.das2009@gmail.com; 3 Mechanical Engineering Department, College of Engineering and Islamic Architecture, Umm Al-Qura University, Makkah 24382, Saudi Arabia; mssoufi@uqu.edu.sa; 4 Department of Production Engineering and Mechanical Design, Faculty of Engineering, Minia University, Minya 61519, Egypt; 5 Department of Production Engineering and Mechanical Design, Faculty of Engineering, Tanta University, Tanta 31527, Egypt

**Keywords:** machinability, cooling-lubrication, SiAlON ceramic, Nitronic 60

## Abstract

The machining of nickel-based super alloys is challenging, owing to the generation of high cutting temperatures, as well as difficulty in maintaining dimensional accuracy and minimizing surface roughness, which compels the use of cutting fluids for reducing these issues due to efficient cooling/lubrication strategies. The present work investigates the comparative performance of four cooling/lubrication techniques: dry cutting, wet, minimum quantity lubricant (MQL) and compressed-air modes in turning Nitronic 60 steel using a new-generation SiAlON ceramic inserts. Several machinability parameters were analyzed for performance evaluation. For this purpose, 16 cycles of turning trials were performed based on Taguchi’s L_16_ orthogonal array experimental design by varying cutting conditions and lubrication modes. MQL exhibits beneficial effects as compared to the other lubrication conditions concerning low cutting force, improved surface finish, decreased cutting temperature, longer tool life, and lower white layer thickness on machined surface. Burr formation on the saw-tooth chip surface, as well as friction, greatly influenced the tool flank wear due to improper cooling and poor lubrication approach in dry, wet, and compressed-air-cooled machining environments in comparison to MQL-machining. From an economical perspective, the tool life in MQL machining improved by 11%, 72%, and 138% in the comparison with flooded, compressed-air, and dry conditions, respectively. The results of the study demonstrate that using the MQL system can help with heat extraction capability, and provide some promising outcomes.

## 1. Introduction

Today, nickel-based alloys are commonly employed for various industrial applications due to their unique properties such as weldability, formability, capability to retain its strength and toughness at elevated temperatures, and excellent resistance to creep, thermal, and corrosion [1]. Owing to its outstanding physical and chemical characteristics, the use of nickel-based alloy is not only bound to the aerospace industry, but it is also extensively used in nuclear power plants, oil and gas power industries, architecture building and construction, transport, food processing equipment in various consumer products, and medical applications.

Machining of hard-to-cut alloys such as titanium alloys and nickel-based super alloys is a challenging and troublesome task owing to the generation of higher cutting temperatures, as well as a difficulty in maintaining dimensional accuracy and minimizing surface roughness [2,3,4,5,6]. To machine the nickel-based super alloys effectively nontraditional machining techniques should be applied or at least machining variables and their range should be carefully selected [7,8]. Such harsh conditions during machining compel the use of cutting fluids for reducing the cutting temperature and deliver sufficient lubrication [9]. In addition, the use of cutting fluid enhances tool life during turning hardened steel in comparison to dry turning [10]. However, the application of adequate amount of cutting fluids using conventional strategy brings extra cost, minimizes productivity, and negatively impacts operator health, as well as enhancing environmental hazards [11]. To overcome these challenges, minimum quantity lubrication (MQL) strategy has been effectively employed by researchers and industries during precise machining of hardened steel [12]. The MQL system efficiently controls the volume of cutting fluid and improves the cutting fluid performance for better penetration in cutting zone [13]. Further, the main issues related to cutting fluid concern ecological, economic and environmental sustainability [14]. This means that the biodegradability of cutting fluid is poor as well, since it is composed of hazardous chemical constituents. However, the use of automotive radiator coolant (a mixture of de-ionized water, glycol) as cutting fluid in machining was acknowledged in past studies due to their non-toxic behavior, lower health and environmental hazards presented, and enhanced sustainability [15]. Therefore, the application of green radiator coolant as a cutting fluid with MQL system unlocks several important domains. Nowadays, production industries are growing on a large scale, and both the State and central government are imposing strict regulations towards green and sustainable manufacturing policies [16,17,18]. That is why several scientists and researchers have been attempted on environmentally sustainable cooling-lubrication systems to enhance the cutting performance and machinability of different hard-to-cut and difficult-to-cut workpiece materials during turning operations (EN-24, EN-31,42CrMo4, 17CrNiMo6, Haynes-25, Ti6Al4V, Inconel 825, 800, 718, AISI 202, AISI 316, AISI 420, AISI 1015, AISI 4140, AISI 4340, AISI 1045, AISI 1060, AISI 52100, AISI D2, D3) [19,20,21,22,23]. In the past, several researches have conducted the application of vegetable-based cutting fluid with MQL, and recommended an improved machining performance in terms of cutting force, tool wear, and surface roughness for MQL-assisted cutting fluid in comparison to dry and wet machining [24,25,26].

Uncoated and coated carbide tools are favored in many cases, while machining superalloy materials. Despite, the performance of these tools is inadequate, particularly at higher cutting speeds. The main reason for this situation is that the extreme temperature that occurs at high cutting speeds weakens the material properties of the tool. Classed among the favorite ways of eliminating this negative is using ceramic tools, which enable working at high cutting speeds due to high thermo-chemical stability, wear resistance, and hardness [27]. However, among two different category ceramics (Al_2_O_3_ based and Si_3_N_4_ based), the silicon nitride-based SiAlON ceramic tool has received considerable attention towards its application as a cutting tool in machining of various hard and difficult-to-cut metals, owing to superior characteristics. 

Based on the open research bibliographies, machining under several lubrication cooling methods [28,29,30] (spray impingement cooling, compressed-air, flooded, dry, and MQL) are available aplenty. However, none of these studies explored in a systematic way, which is required for worthwhile research in terms of uniqueness to consider the physical aspect of machining science. From the open literature, it is clear that many investigators have carried out Al_2_O_3_-based ceramic tools during machining to assess their cutting performance [31,32,33]. However, the use of newer generation silicon-strengthened. SiAlON grade ceramic tool is limited in finish turning, which provides a new avenue of research for machinability enhancement of Nitronic 60 alloy steel [34,35]. To fill the research gaps in consideration of the contributions in last published works, the aims of present work are: (i) to evaluate cutting efficiency of new generation SiAlON ceramic insert; and (ii) to explore a comparative investigation towards machinability enhancement of Nitronic 60 steel by employing various lubrication cooling systems (dry, flooded, compressed-air, and MQL). All of the above contributions offer valuable research, raising the uniqueness of the current analysis.

## 2. Experimental Setup and Procedure

**Figure 1:**
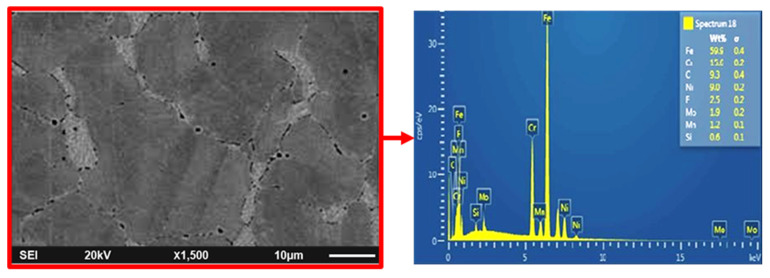
SEM/EDX image of Nitronic 60 steel.

**Figure 2:**
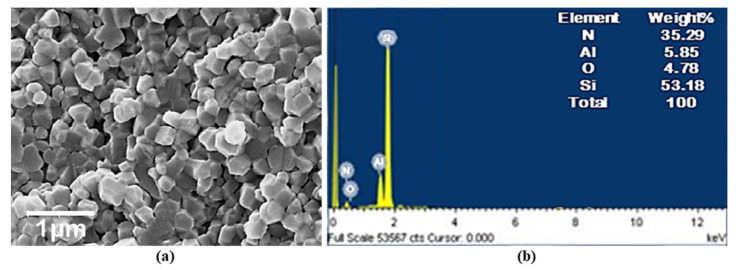
(**a**) Microscopic analysis through SEM and (**b**) EDX ray spectroscopic analysis of SiAlON ceramic tool [34].

In the present investigation, Nitronic 60 steel measuring 50 mm in diameter and 500 mm in length was used as workpiece material for determining machining responses during turning experimentations. Due to high corrosion as well as hot strength resistance, Nitronic 60 has vast applications in various industrial sectors related to automobile, marine, and power generation. The SEM with EDX for Nitronic 60 steel was shown in Figure 1. Table 1 shows the properties of Nitronic 60. The turning operation was accomplished on heavy duty high precision lathe machine (model: NH26) manufactured by HMT Machine Tools Ltd., Bangalore, India. New generation SiAlON ceramic inserts having grade KYS30 and ISO designation SNMG 120,412 manufactured by Kennametal (Bengaluru, India)), was used in the turning of the Nitronic 60 workpiece. This insert was selected in particular because it has a wide application of machining hard and difficult to cut materials used in aerospace industries. The insert possesses effective hardness, wear resistance and toughness. The micrographical analysis of the SiAlON ceramic insert and its elemental analysis was combinedly accomplished using SEM (JSM-6084LV, JEOL, Tokyo, Japan) and EDX facilities. The result is shown in Figure 2. The microstructure of the SiAlON ceramic artistic contained a uniform equiaxed grain morphology with a standard particle size of approximately 0.5–1.0 µm which reflects the constitutionally improved hardness of fine-granulated sialons, providing prominent resistance to material removal from tool substrate. The tool holder specification PSBNR2020K12 was employed in the current study. Green colored ecofriendly automobile radiator coolant (make: Wurth, Maharashtra, India.) was used for both flooded and MQL conditions as a cutting fluid. In MQL machining, the coolant was delivered by regulating the air pressure of 4 bar and 60 mL/h of fluid flow rate. The supply of coolant to the machining zone via nozzle was positioned at an angle of 30°. After each experimental run, the Mitutoyo portable surface roughness tester SJ210 with a cut-off length of 0.8 mm was used for measuring the surface roughness height (Ra) in µm of the finished workpiece. In order to reduce uncertainties due to resumption operations, as well as to achieve the highest precision possible, the measurement of roughness was carried out directly on the machine tool without disassembly. The measurements were repeated three times at three equally spaced locations around the circumference of the machined component, and only the averaged value was considered under each machining condition. Once the surface roughness was measured, to study the aspect of white layer formation, samples were cut from the machined surface with the help of a wire-electrical discharge machine (WEDM). Prior to actual testing, the cold-mounted specimens were polished with different grit sizes of waterproof SiC papers of grade (P600, P800, P1000, P1200, and P1500). Finally, the specimen surface had been polished by velvet cloth with diamond paste of 1 μm size, and lastly by Hifin fluid. Next, the polished surface had been chemically etched with Nital (a solution of ethyl alcohol and nitric acid) for 20 s immersion. The specimen prepared by the above mentioned standard metallographic techniques were used in order to reveal the white layer formation on the machined surface. An advanced optical microscope (model: Axiotech 100HD-3D) developed by Zeiss (Bengaluru, India) was engaged to evaluate tool wear on the flank and rake surfaces of the tool insert after completing each experimental trial. A FLUKE-made (Bengaluru, India) Ti32 infrared thermal imaging camera possessing emissivity value set at 0.96 was employed to monitor the cutting temperature at the interface of the work and cutting edge of the tool. The morphological analysis of machined surfaces and worn out surfaces of the cutting inserts were accomplished via scanning electron microscopy (make: JEOL, Tokyo, Japan; model: JSM-6084LV). 

**Figure 3:**
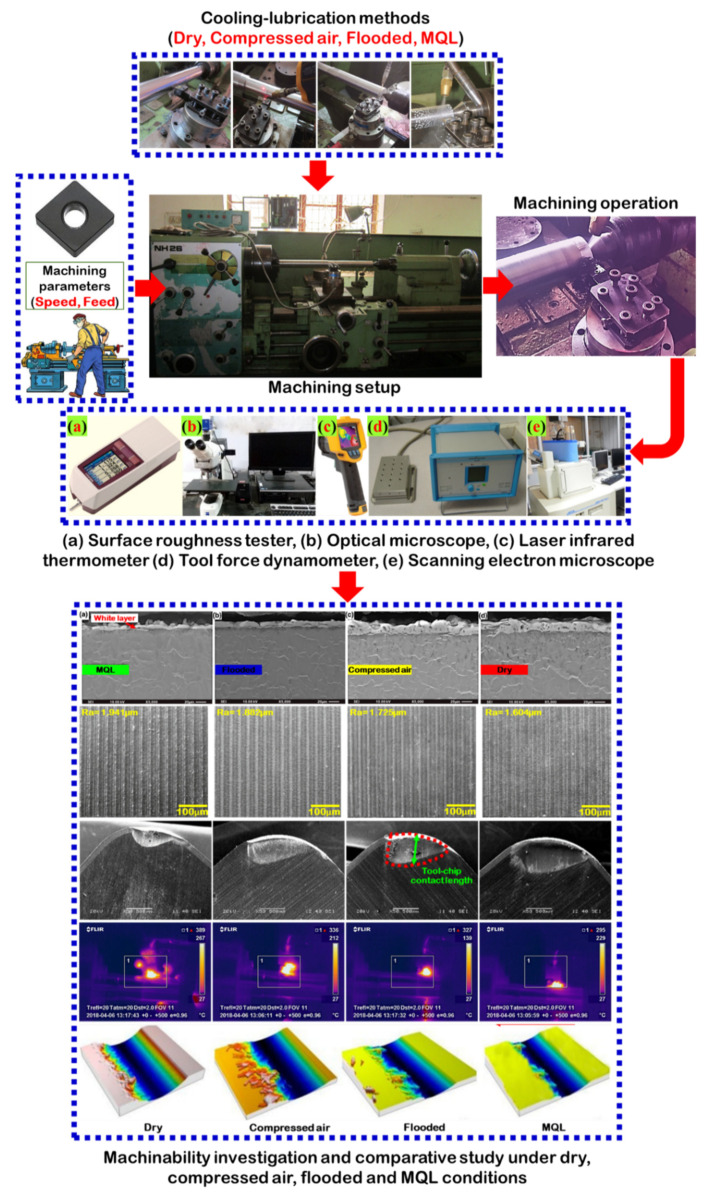
Graphical illustration of experimental setup and discussion of results. Adapted from [34].

Traditional experimental design procedures are very complicated and also difficult to handle, especially where a large number of experiments have to be carried out for a higher number of variables. The Taguchi method is a systematic application of design and analysis for experiments. The experimental design proposed by Taguchi involves orthogonal arrays to organize the parameters affecting the process and the levels at which they should be varied. The advantage of the Taguchi method of experiment is designed to reduce cost and time in machining by selecting the best combination of inputs to produce a product or service. Taguchi’s orthogonal arrays are highly fractional orthogonal designs. These designs can be used to estimate main effects using only a few experimental runs. In this study, three control variables with four levels, such as axial feed (0.24, 0.20, 0.16, 0.12 mm/rev), cutting speed (111, 87, 67, 51 m/min), and lubrication cooling methods (dry, MQL, flooded, compressed-air), were taken into consideration for investigation towards the machinability of Nitronic 60 steel in terms of flank and crater wears, cutting temperature, surface roughness, and cutting force. During machining, the depth of cut was considered constant at 0.4 mm. The selection of different levels of machining parameters were considered with reference to published research work and recommendation of cutting tool manufacturer. The number of machining trials were executed according to L_16_ orthogonal array, considered according to the Taguchi method to execute a minimum experiment number. The cutting conditions employed in each trial are listed in Table 2. Figure 3 illustrates a schematic of the procedures of the experimental work carried out in this study.

## 3. Results and Discussion 

### 3.1. Analysis on Tool Wear

**Figure 4:**
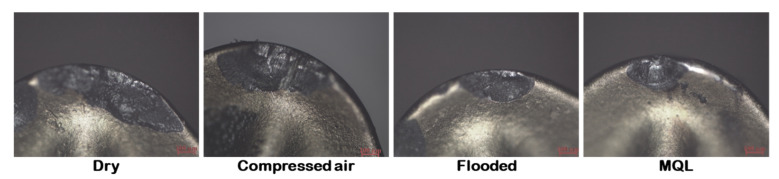
Images of crater wear under several turning environments at v = 67 m/min, f = 0.20 mm/rev.

The optical images of carter wear under various machining environments (dry, compressed-air, flooded, MQL) are shown in Figure 4. Under dry as well as compressed-air-cooled cutting conditions, due to the weak heat transfer capability of cooling medium, an enormous quantity of heat is developed at the interface of work and tool, which is responsible for the development of thermal stress which leads to the starting of cracking on rake face followed by edge chipping. However, due to the surplus availability of coolant at the cutting zone in flooded cooling conditions, a large amount of heat was removed from the cutting zone, thus it effectively minimizes the carter wear by reducing the generation of thermal stress on the tool rake surface. However, tool chipping may be caused by the thermal shocking of using a coolant, especially in flooded condition. In view of MQL condition, as a cooling medium is applied in jet form with high pressure, it competently extracted heat from the cutting region, and a resulting decrease in thermal stress build up in the cutting tool. Thus, less chipping was observed on the tool insert in MQL condition than that of other C/L environments. Still, some edge chipping was observed in MQL condition. 

**Figure 5:**
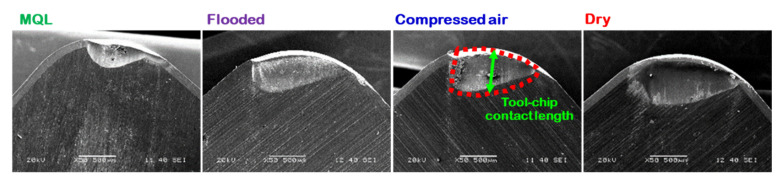
Crater wear due to tool-chip sliding contact length under several turning environments at v = 87 m/min, and f = 0.24 mm/rev.

When machining at high feed rate, continuous chips were produced, and chip flow was almost up curl in nature and thus, producing spiral chips. These chips were in contact on the workpiece surface at their end, and rolled more than one revolution. Due to the end contact of the chips with the workpiece, chips are straightened, and their curvature radius increased. The localized sliding at the tool–chip interface in terms of longitudinal grooves is observed. The longitudinal grooves are shown at the contact area in the direction of chip flow. This localized sliding leads to an irregular slide flow of the continuous chips formed during the process over tool rake surface, thereby producing long tool-chip contact length. Moreover, more tool wear is observed on the rake surface. Figure 5 shows the increment of crater wear due to increased tool–chip contact when the cutting environments changed from MQL to dry condition. Due to effective flushing effect of coolant and controlled of temperature at the machining zone in MQL, a comparatively lesser crater wear followed by tool–chip contact length resulted. 

**Figure 6:**
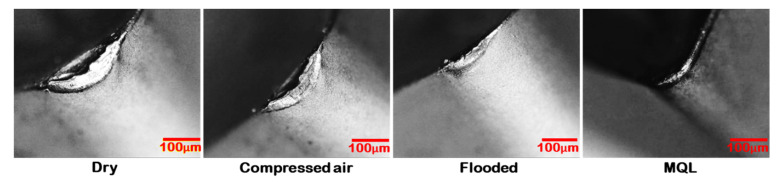
Images of flank wear under various turning environments at v = 67 m/min, and f = 0.24 mm/rev.

**Figure 7:**
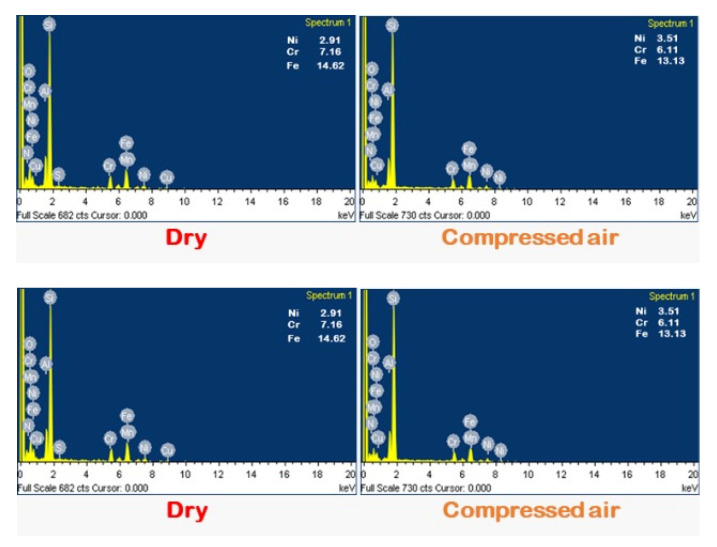
EDS spectrum of tool’s flank surface under various cutting environments at f = 0.24 mm/rev, v = 111 m/min. Adapted from [34].

Figure 6 shows flank wear on the cutting insert in different C/L methods. Maximum wear on the flank face was noticed in the dry cutting environment, whereas it was minimum in MQL cutting. Since coolant is not applied in dry turning, the heat generated during cutting is accumulated at the cutting region, causing a rapid increase in cutting temperature. This high temperature promoted the formation of notch due to high thermal stress and adhesion of the melted chip to the flank surface of the tool. Due to improper lubrication, chipping and abrasion type of wear are also seen on the flank face of the cutting tool. The compressed air-cooled machining gave better results than the dry-cut flank wear due to better heat removal. It is well established that, in convection, more heat can be transferred. However, in provision for MQL condition, due to its effective C/L capacity, slight edge chipping, negligible adhered material, compact notch, and very smooth abrasion marks were noticed, since compressed-air-cooled condition gave better results in comparison to the dry cutting condition. The EDS analysis examined the flank face of SiAlON ceramic tool under different cutting environment, as shown in Figure 7. It is noted that the deposition of work material is dominant in dry machining and minimal in MQL machining. This might be attributed to diffusion of work material into the tool during dry turning, which generates a lot of heat at high cutting speeds. The presence of Cr, Mn, and Fe indicates that particles of the chips get stuck to edges. In the particular spectrum, the deposition of aforesaid elements on the flank surface are collectively 25%, 22%, 13%, and 11%, respectively under dry, compressed-air, flooded, and MQL conditions. 

### 3.2. Analysis on Tool Life

**Figure 8:**
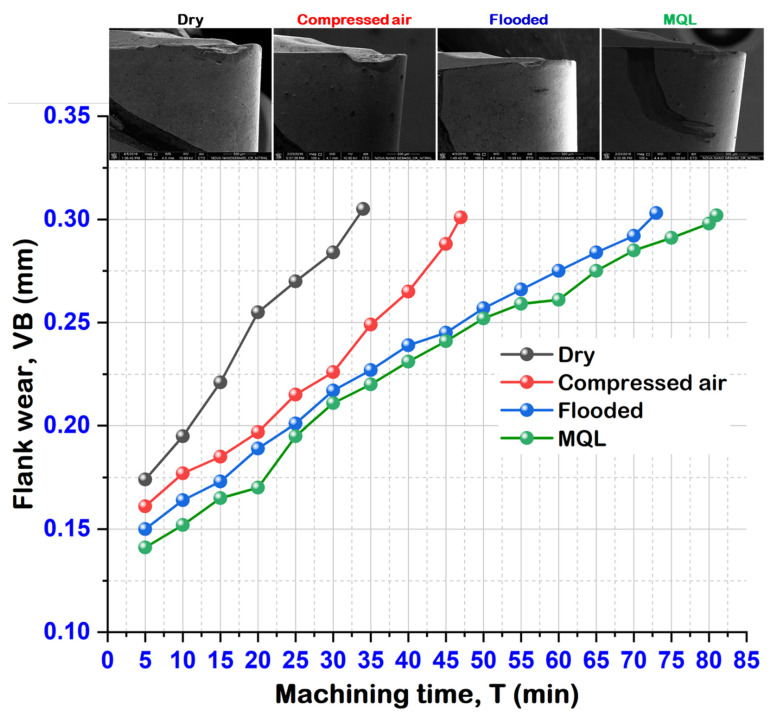
Tool life illustrating the progression of flank wear up to 0.3 mm under various cutting environments at f = 0.24 mm/rev, v = 111 m/min.

Figure 8 illustrates the life of the SiAlON ceramic cutting tool inserts under various lubrication cooling strategies. This analysis considers the ISO 3685 standard to evaluate the tool life based on the machining duration to reach up to the control limit of flank wear (VB) 0.3 mm. Under the aforementioned lubrication cooling methods, various types of wear patterns were observed for tool failure, namely chipping of cutting edge, nose wear, abrasion marks, and notch. However, considerably less damage was observed at the region of main cutting edge under MQL. Results revealed the maximum tool life of 81 min under MQL condition while 73 min, 47 min, and 34 min were achieved in the cases of machining under flooded, compressed-air, and dry environments. From an economical perspective, the tool life in MQL machining improved by 11%, 72%, and 138% more than that acquired under flooded, compressed-air, and dry conditions, respectively. 

**Figure 9:**
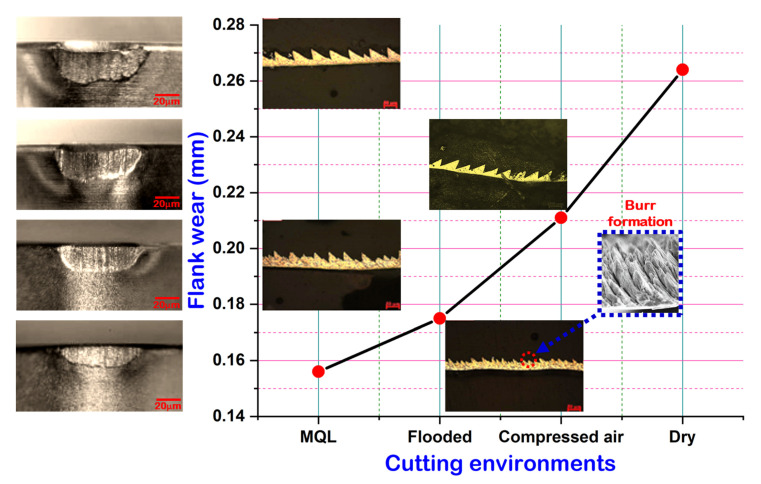
Effect of serrated saw-tooth chip on tool’s flank wear at v = 111 m/min; f = 0.20 mm/rev.

For better illustration, it can be observed from Figure 9 that the development of tool wear is specifically related to the formation of serrated saw-tooth chips. More saw-tooth chips shapes were observed when the cutting environments changed from MQL to dry conditions. Due to the absence of coolant in dry machining, burr formation on the serrated chip was prominent, and occurred as a result of intensive shear bands developed by severe plastic deformation. The abrading nature of burred-serrated edge of the chip develops grooves on the tool’s flank surface due to prominent friction, as well as the temperature at a higher cutting speed. Thus, this resulted in maximum flank wear under dry machining. A similar observation was reported in the work of Kishawy and Elbestawi [36]. Moreover, at high speed, severe abrasion marks are observed under dry and compressed-air conditions. This is attributed to the presence of hard constituents and the heat treatment phase present in the workpiece. Under dry cutting conditions, abrasion with microgrooves, as well as notches, are observed at the flank surface of the insert. However, during machining with MQL, very thin abrasion marks are noticed.

### 3.3. Analysis on Cutting Temperature 

**Figure 10:**
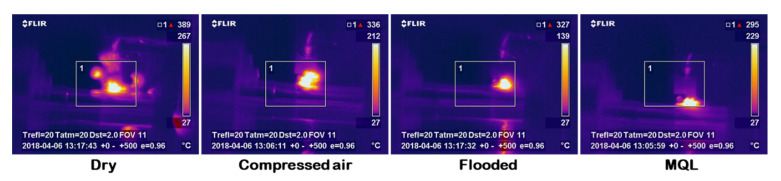
Infrared thermographs show the cutting temperature under different cooling lubrication methods at v = 51 m/min, f = 0.12 mm/rev.

**Figure 11:**
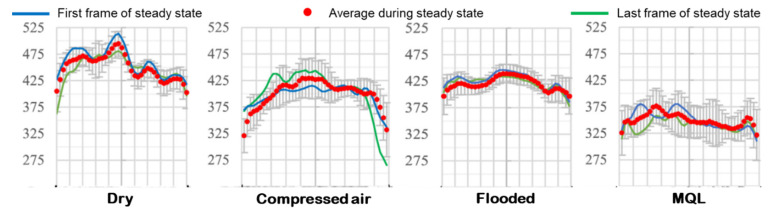
Temperature signal at cutting zone under different cooling lubrication methods (v = 111 m/min, f = 0.24 mm/rev).

Figure 10 depicts IR thermography of the cutting zone temperature in different cooling lubrication methods using an IR thermal imaging camera. Additionally, Figure 11 shows the measurement signal images of the cutting temperature under the influence of various C/L environments. The maximum temperature was observed in a dry cutting environment, whereas it was minimum under the MQL condition. The use of coolant reduces the cutting temperature by providing a highly effective heat extracting medium, as well as lubrication. In addition, the reason for less heat generation under MQL condition is that the friction between the tool–chip is decreased as the contact length between tool and chip is reduced. 

### 3.4. Analysis on Cutting Force

**Figure 12:**
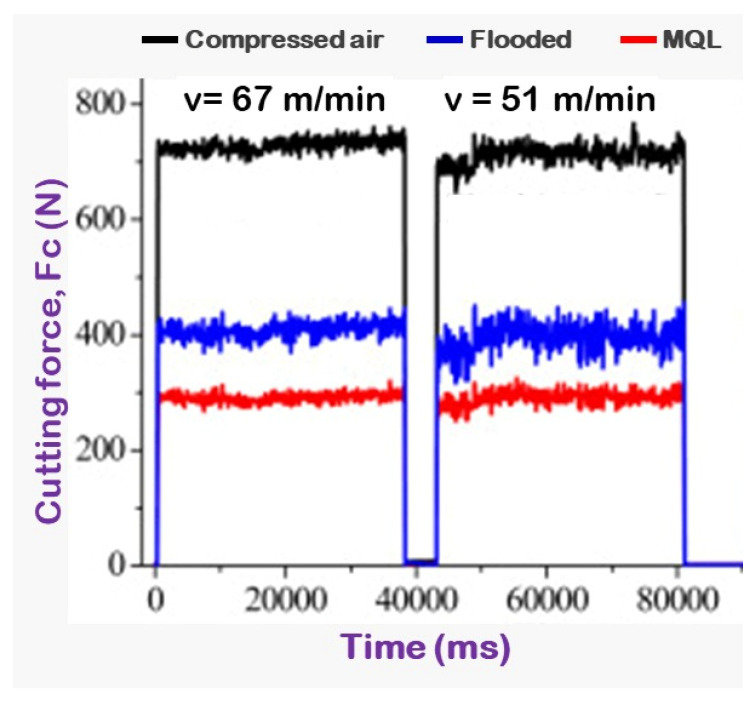
Principal cutting force under compressed-air, flood, and MQL environment at varying speeds. Adapted from [14].

The pictorial view delineates an evaluative study regarding the variation of cutting force under several cutting circumstances, since it vigorously relates the other machining attributes, such as tool life, machining temperature, machined surface morphology, chip morphology, and other surface integrity parameters. From Figure 12, it was found that maximum cutting force was noticed with the compressed-air-cooled cutting condition when compared with flood and MQL environments. The considerable diminishment of cutting force in MQL condition might be due to better cooling and lubrication approaches available in the MQL technique due to suitable positioning of the coolant delivery system. Moreover, minimal flank wear was reported against MQL compared to other machining environments. This might be another reason for the diminishment of cutting force under MQL.

### 3.5. Analysis on Machined Surface Roughness

**Figure 13:**
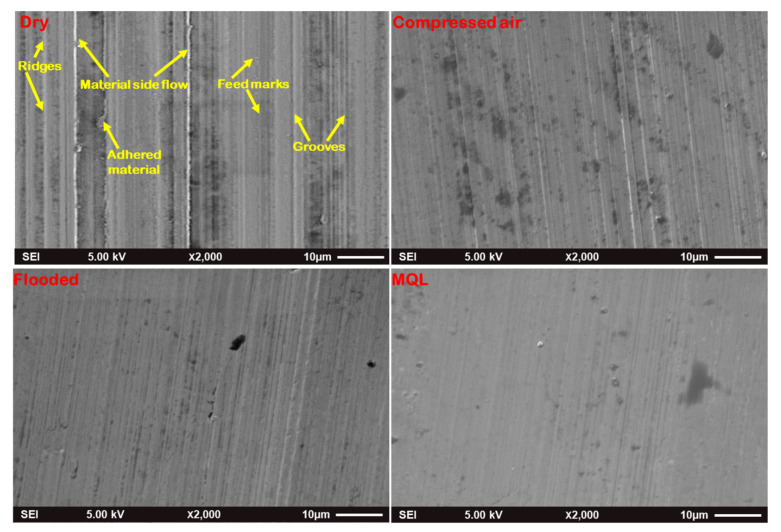
SEM micrographs of machined surface at v = 87 m/min, f = 0.12 mm/rev under different cooling lubrication methods.

**Figure 14:**
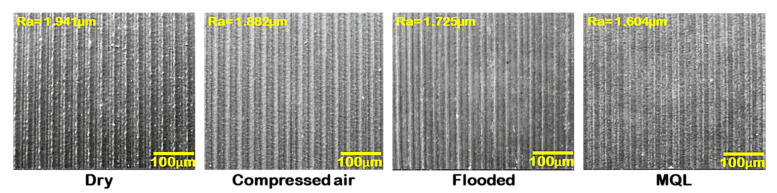
Optical images of machined surface under various cutting environments at v = 111 m/min, f = 0.20 mm/rev.

Figure 13 presents the status of the low-grade hard turned surface concerning feed marks, material side flow, grooves, adhered material, ridges, smeared surface while machining of Nitronic 60. However, machined morphology obtained by MQL-turning resulted better surface quality rather than machining under dry, compressed-air, and flooded environments. Figure 14 depicts the optical images of machined surfaces in precited turning environments. Out of the four precited cutting conditions, the machined surface’s surface topography clearly reflects that turning with MQL presents lower peaks compared to other machining environments. Moreover, it shows reasonably low feed marks and feed mark area expansion to ridges under MQL. As the cutting fluid is absent in dry turning, high friction is generated at the tool–work interface, resulting in wear at the cutting edge followed by increasing cutting force. This increase in the principal cutting force consequently enhances the roughness value of the surface. However, under flooded conditions, due to the presence of abundant cutting fluid, which provides desired cooling and lubrication effect, to a reduction in cutting edge wear followed by improving surface quality is achieved, while in the case of MQL turning, as cutting fluid is applied in spray form, effective cooling is obtained at the friction interface zone due to aerosolization of cutting fluid. Thus, it subsequently protects the tool from thermal stress, and upholds the effectiveness of the cutting insert. In addition, cutting forces also increase relatively less in comparison to other cooling methods. The optimal cutting conditions that minimize the cutting forces and surface roughness could be determined metaheuristic approaches [37,38,39,40]. The process responses could be also predicted using artificial intelligence tools [41,42,43,44].

**Figure 15:**
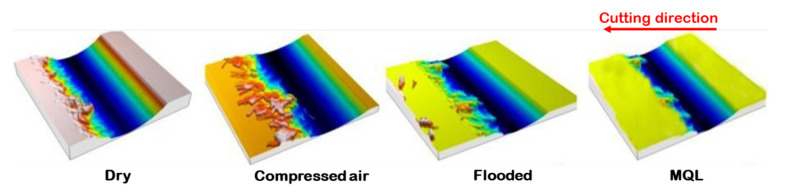
Topography of the machined surfaces due to ploughing effect under different cooling lubrication methods at v = 111 m/min, f = 0.24 mm/rev.

It was deduced that surface finish improved when the cooling lubrication strategy changes from dry to MQL condition. This is because, when machining under a dry environment, the temperature among the tool and work increases at the cutting zone, which develops a severe ploughing effect due to thermal softening of work material. As a result, the burr formation takes place. This refers to material side flow, and thereby supports to material side flow, thus supporting the degradation of machined surface finish under a dry environment compared to other C/L methods. Additionally, there is much more serious squeezing and elastic deformation in the tool–work junction zone under dry environment relative to MQL condition, which induces the material plasticized in the cutting zone flows through the worn trailing edge to the sides of the tool, and hence deteriorates the machined surface finish, as is evident from Figure 15.

**Figure 16:**
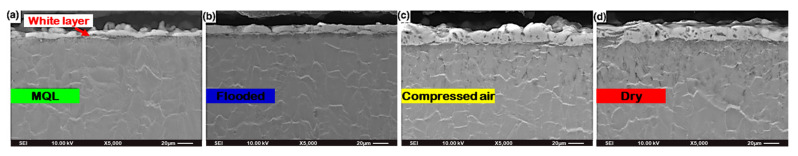
SEM images illustrating the effect of cutting environments on white layer thickness of machined surfaces at f = 0.16 mm/rev, v = 111 m/min for: (**a**) MQL; (**b**) flooded; (**c**) Compressed air; (**d**) Dry.

SEM micrographs of surface and sub-surface regions for machined specimens under C/L environments using SiAlON ceramic inserts are illustrated in Figure 16. It should be noted that the white layer thickness increases when the cutting environments change from MQL to dry. During machining, white layer formation depends upon three major factors (rapid heating and quenching, plastic deformation, and surface reaction) [45]. As discussed earlier, the temperature will increase at the machining zone with speed in dry-cut conditions due to ineffective heat transfer and severe shear deformation. This is the reason for the increment of white layer thickness. Additionally, the present investigation reveals that the development of flank wear is obtained at the highest cutting speed under the dry condition, which sets out the prominent white layer thickness. This could be the reason why the rate of heat dissipation is not properly transferred to the tool substrate, resulting in high temperature at the tool–work interface, for which heat-affected zone is increased, and results in increased white layer thickness. 

## 4. Conclusions

This article presented the performance evaluation of various machining environments (dry, compressed-air cooled, flooded, and MQL) in finish turning of austenitic stainless steel Nitronic 60 with new-generation SiAlON ceramic tools in terms of surface roughness, cutting force, cutting temperature, tool wear, and tool life. Based on the experimental analysis, it is seen that machining under MQL performed comparatively better than the three pre-cited C/L methods. It was observed that, as under MQL conditions, cutting fluid is applied in spray jet form, it effectively removed heat and dewberries from the cutting zone, and outperformed the other methods regarding (i) low cutting force, (ii) improved surface finish, (iii) decrease cutting temperature, (iv) longer tool life, and (v) lower white layer thickness on machined surface. The machined surface morphology explained the status of the low-grade turned surface concerning feed marks, material side flow, grooves, adhered material, ridges, and smeared surface during machining of Nitronic 60. Furthermore, results revealed the maximum tool life of 81 min under MQL condition, while results of 73 min, 47 min, and 34 min are achieved in the cases of machining under flooded, compressed-air, and dry environments. From an economical perspective, the tool life in MQL machining improved by 11%, 72%, and 138% more than that acquired under flooded, compressed-air, and dry conditions, respectively. In comparison to other cooling conditions, the abrading nature of burred-serrated edge of saw-tooth chip with fervid shear bands observed in dry cutting develops prominent grooves on the tool’s flank surface due to influential friction, as well as temperature at a higher cutting speed. The effective flushing effect of coolant and controlled of temperature at the cutting zone under MQL resulted in a comparatively lesser crater wear followed by tool–chip contact length. The results of the study demonstrate that using the MQL system can help with heat extraction capability and provide some promising outcomes. Based on comprehensive experimental research, new-variety SiAlON ceramic tools can be competently and productively utilized for the machining of austenitic stainless steel under ecologically cognizant minimum quantity cooling lubrication conditions, and is the most accepted in industrial applications.

## Figures and Tables

**Table 1:** Properties of Nitronic 60 [34].

Density	Poisons Ratio	Tensile Strength	Modulus of Elasticity	Thermal Conductivity	Vickers Hardness
7622 kg/mm^3^	0.298	395 MPa	200 GPa	51.9 W/mK	115 HV

**Table 2:** Experimental design layout.

Test No.	Cutting Speed, v (m/min)	Feed Rate, f (mm/rev)	Cutting Environments
1	51	0.12	Dry
2	51	0.16	Compressed-air
3	51	0.20	Flooded
4	51	0.24	MQL
5	67	0.12	Compressed-air
6	67	0.16	Dry
7	67	0.20	MQL
8	67	0.24	Flooded
9	87	0.12	Flooded
10	87	0.16	MQL
11	87	0.20	Dry
12	87	0.24	Compressed-air
13	111	0.12	MQL
14	111	0.16	Flooded
15	111	0.20	Compressed-air
16	111	0.24	Dry

## Data Availability

Data is contained within the article.
